# Mechanical and frictional properties of coconut husk powder reinforced polymer immersed in a simulated acidic medium for oil/gas applications

**DOI:** 10.1016/j.heliyon.2024.e25026

**Published:** 2024-01-22

**Authors:** David O. Obada, Kazeem A. Salami, Ayodeji N. Oyedeji, Obinna A. Osuchukwu, Jimoh Abass, Christian Ogwuche, Naresh D. Bansod, Michael I. Ubgaja, Ibrahim U. Ibrahim, Bello Abdulkareem, Rabiu K. Olawale, Luqman K. Abidoye

**Affiliations:** a Department of Mechanical Engineering, Ahmadu Bello University, Zaria, Nigeria; b Africa Centre of Excellence on New Pedagogies in Engineering Education, Ahmadu Bello University, Zaria, Nigeria; c Multifunctional Materials Laboratory, Shell Office Complex, Department of Mechanical Engineering, Ahmadu Bello University, Zaria, Nigeria; d Department of Chemistry, Mahatma Gandhi Arts, Science and Late N.P. Commerce College, Armori (M.S.), India; e Department of Polymer Technology, Nigerian Institute of Leather and Science Technology, Zaria, Kaduna State, Nigeria; f Department of Chemical Engineering, Osun State University, Osogbo, Nigeria; g Department of Mechanical Engineering, Bayero University, Kano, Nigeria

**Keywords:** Low-density polyethylene, Acid ageing, Mechanical properties, Corrosion, Frictional behaviour, Oil and gas, Water absorption

## Abstract

Polymeric materials are constantly exposed to aggressive environments, negatively impacting their mechanical and chemical properties. In salt, acid, or alkaline solutions, polymer materials degrade due to surface flaws, microcracks, or other irregularities. For the first time, this study considers the behaviour of coconut powder/coir-reinforced synthetic LDPE hybrid composite immersed in an aggressive (acidic) medium for 15, 30 and 45 days. The structural, mechanical, and frictional behaviour of the developed coir/coconut husk powder/LDPE hybrid composites were measured after ageing in hydrochloric acid (HCl) as potential materials for oil and gas applications. From the XRD patterns, the prominent reflections in the control samples increased with the acid ageing days, while less prominent reflections characterized the hybrid composites. The hardness of the reinforced samples immersed for 30 and 45 days (30B and 45A) showed the highest values of 0.28 Hv, while the control samples immersed for 15 days had the least hardness. The reinforced samples immersed for 15 and 30 days (15B and 30B) showed the lowest and highest fracture toughness, respectively. The control samples were observed to absorb little water after immersion for 144 h. The result showed that although the reinforced hybrid composites showed better mechanical properties, with an increase in the days of immersion in an aggressive medium, the properties became compromised compared to the un-reinforced samples. Hence, the applications of the produced reinforced polymers in the oil and gas industries may be limited.

## 1 Introduction

Studies have drawn more interest in the use of natural plant fibres as reinforcing materials in composites due to certain desirable properties [1,2]. A contributing factor to this increasing interest is the quest to stem the tide of environmental pollution and degradation. For instance, manufacturing industries like construction and automotive industries continually search for materials that can substitute for non-biodegradable materials like glass fibre for reinforcing polymeric materials [3]. Its lightweight, low cost, low density, biodegradability and many others are the benefits of naturally sourced fibres over glass fibres [4,5]. Fibres in polymer matrix serve as load-bearing elements, providing the needed strength while maintaining fibre alignment. The fibre included has shown improvement in the properties of low-density polyethylene polymer composites leading to its suitability for various applications [6].

There are many disadvantages to using metals like steel in the oil and gas industry. For instance, the exposure of these materials to a corrosive environment makes them prone to corrosion [[7], [8], [9]]. The use of organic inhibitors has proven to be an alternative, but the harmful effect on the environment and humans has been a great concern [10]. Hence, research into using polymeric composite materials for applications in the oil and gas industry might suffice as an alternative to metals. However, during operation, the polymeric materials might be subjected to load, friction and wear due to the combination of the working environment and the applied loads, causing stress corrosion cracking. Several factors can affect the polymers’ application, including mechanical properties, molecular structure, and viscoelastic behaviour [11,12]. A neat polymer may have inadequate mechanical strength for industrial applications. Hence, reinforcing the polymer with high-strength fibres could enhance the mechanical properties making it suitable for numerous applications ranging from sports equipment to the aerospace industry [9]. However, polymeric materials like low-density polyethylene (LDPE) have poor wettability leading to poor bonding with the fibre and void inclusions. These are key challenges in fabricating fibre-reinforced polymer materials [13,14]. Several studies are currently ongoing on improving fibre/matrix bonds through numerous surface treatments and modifying the matrix to improve the composite characteristics. In addition, industries are constantly looking for durable polymer materials with low wear rates and high mechanical strength [15].

In light of these challenges, ongoing research aims to enhance the mechanical properties of polymers for diverse applications [16]. One approach involves incorporating fillers like sawdust, which has shown promise in improving tensile strength and elastic modulus up to a certain threshold but can lead to interfacial bonding issues at higher concentrations [17]. Similarly, studies have explored using laterite as a filler resulting in improved mechanical properties and enhanced fire resistance in polyester composites [18]. The application of polymers such as epoxy-resin has also been used to develop materials with exceptional mechanical robustness and exhibited prolonged anti-corrosion durability in a corrosive environment, effectively impeding access to corrosive electrolytes and significantly improving the coating system's service life compared to other polymer coatings [19]. Similarly, Tian et al. [20] discovered that multi-fillers reinforced epoxy composites exhibit promising long-term hygrothermal resistance as anti-corrosion and anti-wear coatings suitable for harsh environmental applications.

Polymeric materials are mostly exposed to aggressive environments that negatively impact their mechanical and chemical properties [21]. The degradation of the polymer materials occur in salt, acid or alkaline medium through either surface, micro-cracks or other imperfections [8,9,22,23]. Naturally, water intake by a fibre-reinforced polymeric material causes physical degradation like swelling and hydrolysis. Swelling causes de-bonding through the different expansion coefficients between the polymer and the fibre. Also, the dissolution of linking agents within the polymeric chains is possible, causing a reduction in the mechanical strength [24]. In contrast, hydrolysis causes the bond to weaken, causing a loss of the matrix's and fibre's adherence. The change of pH in water might alter the chemistry of the polymer materials [25] through the creation of cracks, degradation, leaching and de-bonding. Several studies have been conducted to study the effect of environmental conditions on the degradation of polymeric materials [[26], [27], [28]].

Consequently, this study aims to address a growing need for a comprehensive research into polymer behaviour under aggressive conditions. The widespread use of sachet packaging made from LDPE has increased the availability of LDPE polymer, especially in developing countries [13]. The availability of LDPE polymer from the sachet water industry has implications for its potential use in the oil and gas sector. LDPE, known for its flexibility, chemical resistance, and low moisture absorption can find applications in the oil and gas industry [22]. While glass fibre-reinforced plastics offer high strength, their applicability is constrained by their relatively high production costs [25]. Natural lignocellulose materials like coconut shell and shell powder have emerged as compelling options for reinforcing plastics owing to their exceptional attributes such as high strength and modulus [22].

Therefore, this study investigates the performance of a novel coconut powder/coir-reinforced synthetic LDPE hybrid composite immersed in a harsh acidic medium for extended periods (15, 30, and 45 days). The focus was on evaluating these hybrid composites’ mechanical and frictional characteristics after exposure to hydrochloric acid (HCl). This research offers a unique contribution to the field as it explores the behaviour of this specific hybrid composite in an acidic environment, providing valuable insights for potential applications in the oil and gas industry.

## 2 Research methodology

### 2.1 Materials

LDPE, a material for packaging sachet water, was obtained from a dumping site on campus at Ahmadu Bello University, Zaria, Nigeria. The coconut coir/fibre was obtained from a Zaria farmers’ market, and the HCl used for the acid ageing experiments was purchased from Cardinal Chemicals, Zaria.

### 2.2 Material preparation and hybrid composite fabrication

The coconut husk fibre was washed to remove impurities and then dried at room temperature. The water resistance properties and cellulose content were improved through alkali treatment using sodium hydroxide (NaOH). 5 wt% of NaOH was mixed with an aqueous solution (concentration of 10 M) to eliminate the residual alkali in the coconut husk fibre for 30 min. This process improves the surface roughness, resulting in improved fibre-matrix compatibility and high mechanical strength [29]. The fibre was sun-dried for 72 h to remove the moisture content. Some parts of the coconut husk fibre were milled to convert the fibre to powdery form (sieved with 425 μm mesh), while the plastic crusher was used to shred the low-density polyethylene (LDPE). The samples were mixed homogeneously using the composition: 70 wt% LDPE, 15 wt% coconut fibre and 15 wt% coconut shell powder, as supported by a previous study conducted by Obada et al. [22]. The mixture was heat-treated using a two-roll mill for 30 min at 180 °C then thermoformed with a hot-pressing machine. The fabricated hybrid composites were acid-aged by immersing the fabricated composite in HCl (pH value: 2.2) for the different ageing intervals of 15, 30 and 45 days. The designation of the composites is given in Table 1.

**Table 1:** Designation of the composites.

Composite Nomenclature	Representation
15A	15 days ageing of LDPE (100 wt%),
15B	15 days ageing of Coir/coconut husk powder/LDPE hybrid composites
30A	30 days ageing of LDPE (100 wt%),
30B	30 days ageing of Coir/coconut husk powder/LDPE hybrid composites
45A	45 days ageing of LDPE (100 wt%),
45B	45 days ageing of Coir/coconut husk powder/LDPE hybrid composites

### 2.3 Hybrid composite characterization

The phases in the hybrid composite were elucidated using an X-ray Diffractometer (Rigaku Miniflex Diffractometer) equipped with a copper tube powered by a 40 kV voltage and a 30 mA current with an initial scan covering 25° to 60°. The XRD patterns were then matched with the ICCD databases using the X'Pert high-score software, and the crystallinities of the samples were determined. Scanning Electron Microscopy (SEM) technique was used to examine the microstructure of the hybrid composites. Before the analysis, the samples were cut into small pieces and coated with a thin layer of gold using a sputter coater to enhance conductivity and prevent charging during imaging. The coated samples were then loaded onto the stage of a high-resolution SEM machine. The SEM machine was operated at an accelerating voltage of 20 kV, and the imaging was performed at a magnification of 500X.

### 2.4 Vickers micro-hardness measurements

Vickers micro-hardness measurements of the samples were performed using a diamond indenter on a Vickers hardness tester (MHV1000Z) with a 300 g load and 10 s loading time. Three (3) different indents were made on each sample to measure the hardness values. The average hardness was then calculated, with the inclusion of an error bar to visually represent the range of variation in the values [30,31]. The test was conducted on the samples before and after the ageing period, and a comparison was made. The fracture toughness (K_1c_) was determined through the application of Eq (1) [32,33].

1 $$K_{lc}=0.016\times {\left(\frac{c}{a}\right)}^{-1.5}\times H_{v}\times {\left(a\right)}^{0.5}$$

Where l is the crack length from the indented centre to the crack tip (mm), 2c = 2 (a + l) in mm, *H*_*v*__*is the*_ Vickers hardness (MPa), and a is the half diagonal length (mm).

### 2.5 Frictional properties evaluation

The friction evaluation was determined with a pin-on-disc type tribometer (DRTB, 70090). A pre-rubbing process was carried out for full contact between the disc surfaces and the pin. The tests were carried out on each test sample after they had been thoroughly cleaned at room temperature (25 ± 5 °C) and in an environment with a relative humidity of 50 ± 10 %. The samples were subjected to a normal load of 5 N, and the disc rotated at a speed of 10 cm/s across a sliding distance of 15.71 m. The test period was also set to 300 s, and the test radius was set to 5 mm. A lower test speed and shorter test period were chosen to avoid excessive wear on the surface and temperature rise because this study concentrated on the friction behaviour at the start of sliding. The friction test parameter settings were the same for each sample to ensure the comparability of test findings from various samples [34,35].

### 2.6 Water absorption test

The BS EN ISO 62:1999 standard was used to examine the water absorption impact on the control and acid-aged hybrid composites. The samples were completely immersed in water until completely saturated, and the weight gained was measured every 24 h. The excess water was soaked out before taking the weight. The percentage of water absorption (W_t_) was calculated using Eq (2).

2 $$W_{t}=\frac{W_{2}-\;W_{1}}{W_{1}}\;X\;100$$

Where W_1_ and W_2_ were the weights of the dry and wet samples, respectively.

## 3 Results and discussion

### 3.1 XRD analysis of the hybrid composite before and after acid ageing

**Fig. 1:**
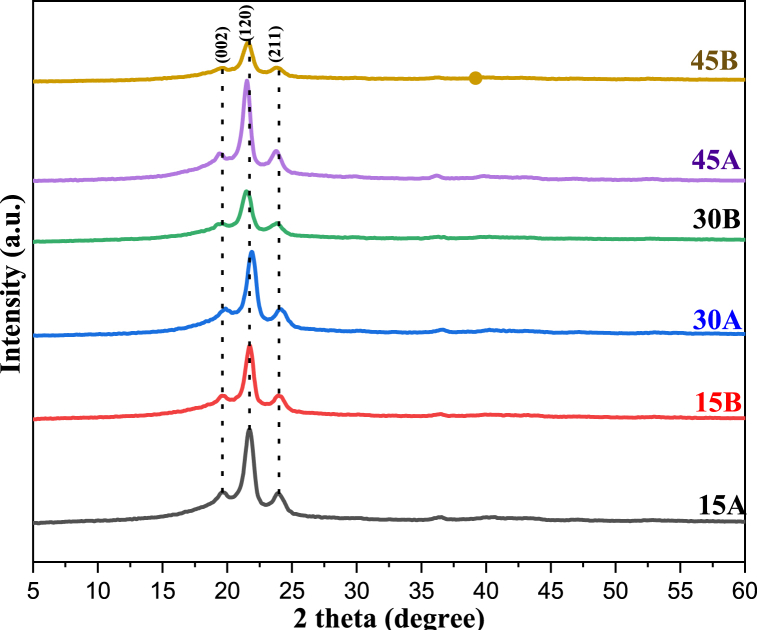
XRD patterns of the acid-aged samples.

**Fig. 2:**
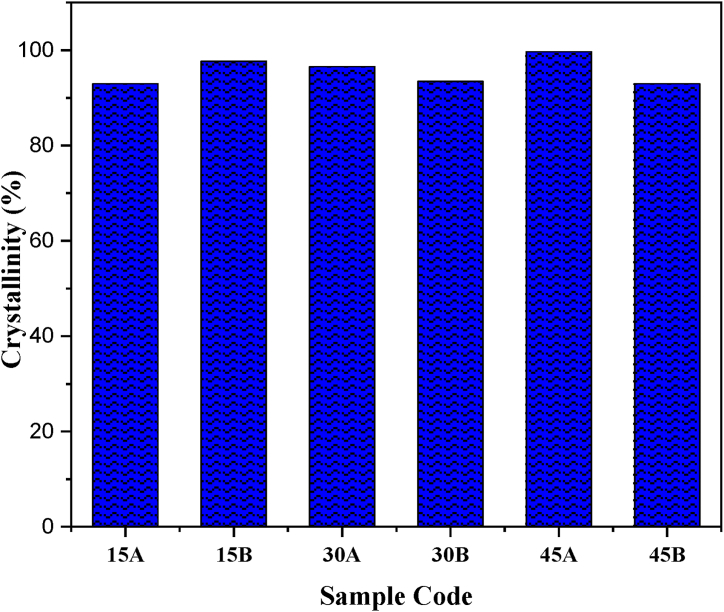
Crystallinity data of the acid-aged samples.

The X-ray diffraction analysis was employed to investigate the effect of acid ageing on the polymer samples, as depicted in Fig. 1. The distinctive reflection of LDPE attributed to the (120) Miller plane, is at 21.4°. Upon exposure to the acidic medium (HCl), the reflections of the pure LDPE sample increased with the number of ageing days, suggesting an enhancement in the polymer chain. This observation aligns with the findings of Eli et al. [36], who analyzed the degradability enhancement of PLA in acidic stearate. Contrarily, the prominent reflections of the composite (LDPE + coir) decreased with an increase in the number of ageing days. This ageing effect appears to diminish the reflection intensity, thereby allowing more amorphous regions within the crystal structure, similar to the observations made by Mateker et al. [37] in their study on organic solar cell materials. This amorphous transformation also reduced the crystallinity, as shown in Fig. 2. It is evident from Fig. 2 that the composites’ degree of structural order may have been influenced, which might impact the mechanical properties and coefficient of friction. This is in line with the study by Boey et al. [38], who observed that crystalline cellulose consists of chains with an orderly molecular arrangement, and amorphous cellulose consists of random arrangements. However, acid ageing increases the peak intensity for control samples, leading to more oriented chains and a possible increase in mechanical properties [38].

From the XRD patterns, the crystallinity of the samples was calculated, and the results show that 15A, 15B, 30A, 30B, 45A and 45B have crystallinity values of 92.9, 97.7, 96.6, 93.5, 99.7 and 93.0 %, respectively. Generally, highly crystalline samples have good mechanical properties [39] since the chains are more aligned. An increase in the ageing interval resulted in decreased crystallinity of the reinforced samples. This could be ascribed to the de-bonding between the fibre and the polymer matrix. However, the crystallinity increases for the control samples, which implies that the acid ageing might have improved the intermolecular bonds [22,40].

### 3.2 Mechanical properties evaluation

**Fig. 3:**
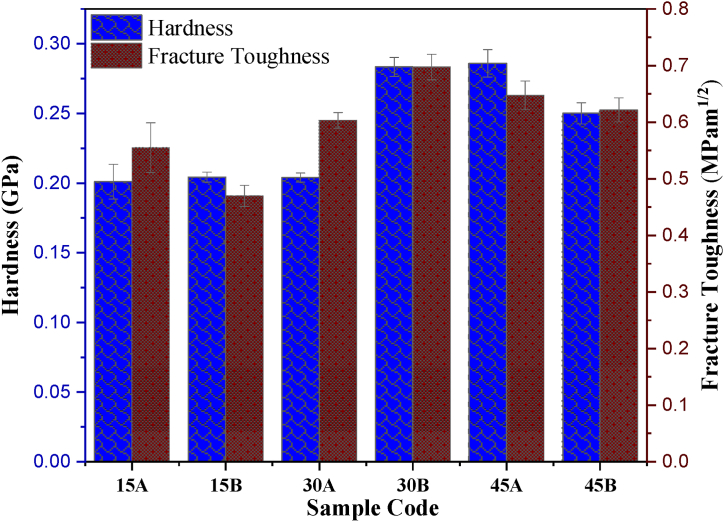
Hardness and fracture toughness data of the acid-aged samples.

The evaluation of the mechanical properties of the polymer samples (15A, 15B, 30A, 30B, 45A, and 45B) is depicted in Fig. 3. For the control samples (samples without reinforcement), a hardness of 0.20 GPa was recorded after 15 days of acid ageing. This hardness increased slightly to 0.204 GPa after 30 days, and a more significant increase was observed after 45 days, with an approximate increment of 20 % (0.25 GPa). The reinforced samples also exhibited a hardness of 0.20 GPa after 15 days of immersion. After 30 days of immersion, however, the hardness increased to 0.28 GPa. The observed increment in hardness is attributed to the extended acid exposure, a trend that aligns with the study by Obada et al. [22]. The possible explanation for this could be that the increase in ageing days led to a larger chain formation, thereby significantly enhancing the polymer strength. This suggests that acid exposure induces changes in hardness, a result that shows a similar trend with Banna et al. [21]. The surge in hardness can be associated with the rise in the degree of crystallinity [31,41,42]. However, a decrease in hardness was observed for the reinforced sample aged for 45 days. This reduction could be attributed to the irregular arrangement in the polymer chains and de-bonding due to van der Waals forces.

**Fig. 4:**
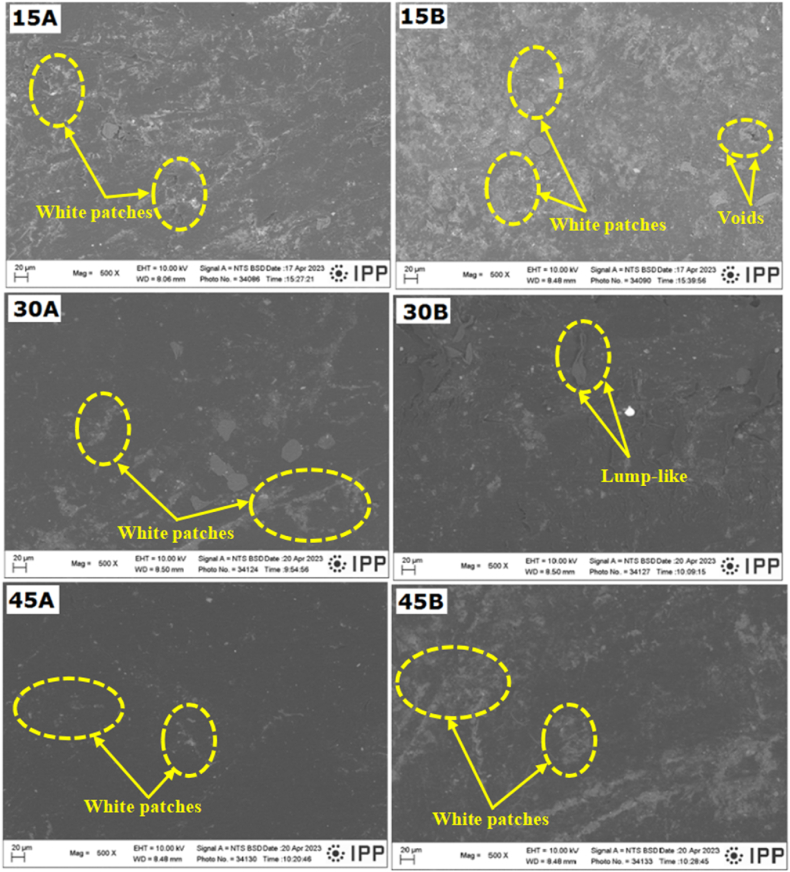
Micrographs of the acid-aged samples.

The fracture toughness obtained for the first 15 days shows a decline from 0.55 to 0.470 MPam^1/2^ after reinforcing, representing an 18 % decrease. This decrease might be ascribed to the irregular or loosely bonded chains, which allow the chains to move easily, leading to a decline in the material's crack resistance. The fracture toughness increased when immersed for 30 days (0.60 and 0.70 MPam^1/2^) for 30A and 30B samples, respectively. For 45 days, the fracture toughness for 45B decreased to 0.6 MPam^1/2^. A further explanation for the enhancement in mechanical properties (hardness and fracture toughness) for the 30B sample is illustrated in the SEM micrographs, as shown in Fig. 4.

### 3.3 Microstructural analysis of the hybrid composite before and after acid ageing

The result of the SEM analysis of the composite before and after acid ageing is illustrated in Fig. 4. White patches were observed for unreinforced samples, but the patches were reduced with increased acid ageing, which is associated with surface roughness. This can be attributed to changes in the pure LDPE's molecular characteristics (intermolecular bonding) [22]. The 45A sample had very few white patches, which may have resulted in its improved mechanical properties comparatively. On the other hand, the white patches observed in the 15B sample were reduced in samples 30B and 45B, with lump-like patches (entanglement) noticed in the 30B sample, an indication of its higher mechanical properties, comparatively. An increase in hardness has been reported by Obada et al. [14] and Banna et al. [43] when polymeric samples were acid-aged due to the chains of the polymeric samples becoming larger and entangled, giving strength to the polymer. The reduction in the lump-like microstructure noticed on the 45B sample may be responsible for the drop in mechanical properties. From the perspective of the swelling of the hydrophilic fibres in the hybrid composites (due to the water absorption ability of the acid-aged hybrid composite samples), some voids and pores are noticeable within the samples with increasing acid ageing time for the reinforced composite variants (15B, 30B and 45B). This observation is consistent with the increased water absorption ability (Fig. 6) observed in the hybrid composites after acid ageing [34].

### 3.4 Coefficient of friction (CoF) evaluation

**Fig. 5:**
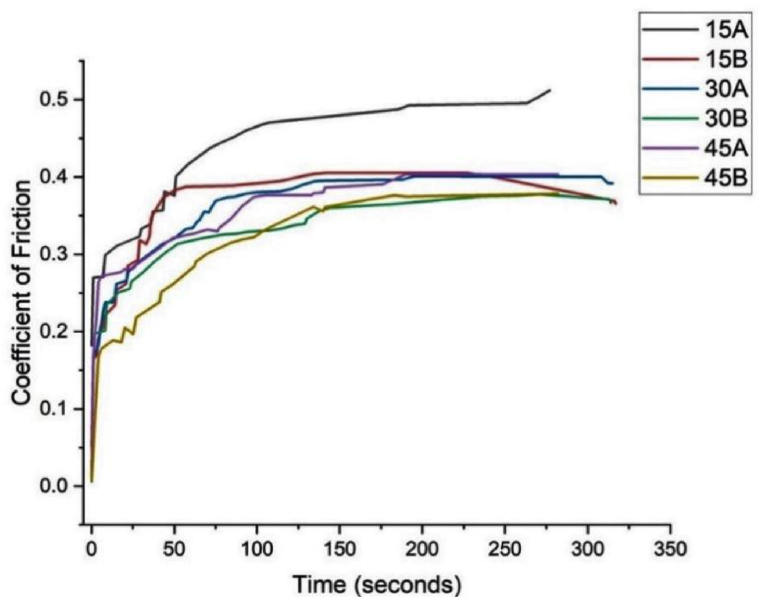
Coefficient of friction (CoF) data of the acid-aged samples.

Fig. 5 presents the coefficient of friction of the polymer samples (15A, 15B, 30A, 30B, 45A, and 45B) as a function of time under a normal load of 5 N. The friction coefficient initially increases from 0 to a peak value, which is referred to as the static friction coefficient. After reaching this peak, it stabilizes, denoting the dynamic friction coefficient. As observed from Fig. 5, the coefficient of friction increases to 0.269, 0.205, 0.182, 0.20, 0.263, and 0.167 for samples 15A, 15B, 30A, 30B, 45A, and 45B, respectively, after the rubbing period. The rise and subsequent decline in the coefficient of friction of the samples could be attributed to the presence of foreign materials (e.g., moisture, oxides, etc.) on the polymer materials during the initial rubbing stage [33,[42], [44]]. Once the initial rubbing removes these foreign materials from the surface of the polymeric materials, the adhesive force at the contact surfaces increases, leading to a ploughing effect [35,45]. In most cases, the frictional force increases with rubbing time until a steady-state value is attained, allowing for a constant value for the coefficient of friction throughout the test time. Generally, acid-aged reinforced samples exhibited lower CoFs compared to the unreinforced samples. The pores and voids caused by fibre pull-out during acid ageing may have facilitated more moisture/liquid intake, thus contributing to the reduced CoFs [33,46].

**Fig. 6:**
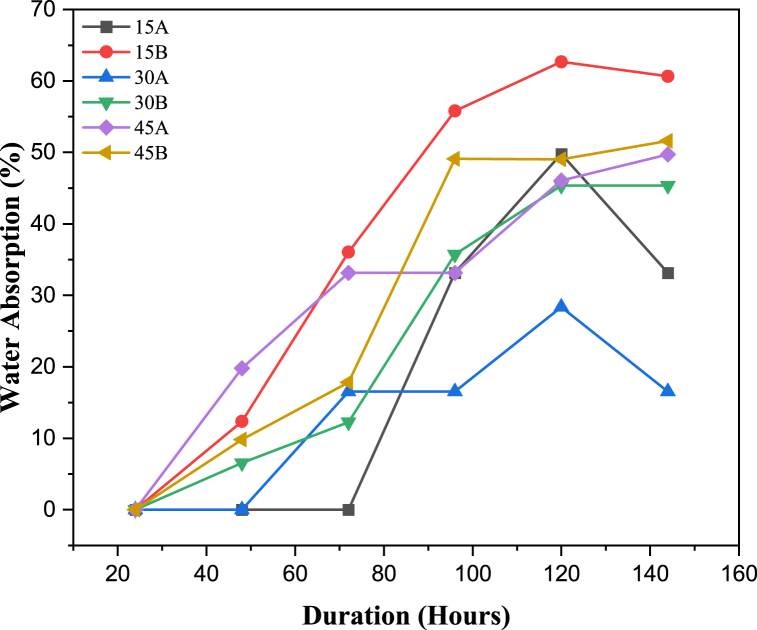
Water absorption data of the acid-aged samples.

### 3.5 Water absorption

The water absorption test was examined for 144 h. Fig. 6 shows that the weight gain by the hybrid composite samples increased more over time during immersion. This is consistent with the findings of Majeed et al. [47], who observed similar behaviour in nanoclay/natural fibres filled hybrid composites. This increase in weight for the reinforced polymer variants can be attributed to the swelling of the coir within the matrix and the formation of voids during acid ageing due to fibre pull-out, a phenomenon also observed by Gholampour et al. [48] in short sisal/glass hybrid fibre-reinforced low-density polyethylene composites. However, the water absorption tendencies of the samples are comparably low generally, with the 15B sample absorbing water the most. This low water absorption of the samples could be attributed to the hydrophobic nature of the LDPE polymer [49]. This aligns with the findings of Linda Bih et al. [34], who noted the hydrophobic nature of polymer matrix-natural fibre composites. The observed behaviour of the hybrid composite samples in the water absorption test could also be influenced by the properties of the natural fibres. As Obada et al. [14] noted, the properties of natural fibre-based composites can vary significantly depending on the plant source and species, among other factors.

## 4 Conclusion

This research investigated the effect of acid ageing for a duration of 15, 30 and 45 days on developed neat LDPE samples and coir/coconut husk powder/LDPE hybrid composite in terms of its mechanical and frictional characteristics. The following results were drawn from the study:

1. The reflections of neat LDPE samples increased with an increase in the number of ageing days, indicating an increase in the polymer chains. However, the prominent reflections of the composite decreased with an increase in the number of ageing days.
2. For the hardness test, the reinforced samples immersed for 30 and 45 days (30B and 45B) showed the highest hardness of 0.28 Hv, while the control samples for 15 days (15A) showed the lowest hardness of 0.20 Hv. The reinforced samples immersed for 15 and 30 days (15B and 30B) showed the lowest and highest fracture toughness, respectively.
3. The swelling of the hydrophilic fibre combined with fibre pull-out created voids within the matrix of the reinforced composite variants and increased the water absorption ability of the acid-aged hybrid composite samples.

The primary limitation of this study is the relatively short duration of the acid ageing tests, which were conducted for 15, 30, and 45 days. While these time frames were selected to capture initial changes in the hybrid composite's properties, they do not provide insights into the long-term performance of the polymeric composites under acidic conditions. Future research could focus on exploring other natural fibres for reinforcement and investigating the effects of other aggressive environments on the properties of these hybrid composites. Additionally, the study of the long-term effects of acid ageing on these composites could provide valuable insights into their potential for use in various industrial applications.

## Data availability

Data will be made available on request.

## Additional information

No additional information is available for this paper.

## CRediT authorship contribution statement

**David O. Obada:** Writing – review & editing, Writing – original draft, Supervision, Methodology, Formal analysis, Data curation, Conceptualization. **Kazeem A. Salami:** Writing – original draft, Formal analysis. **Ayodeji N. Oyedeji:** Writing- review & editing, Formal analysis, Data curation. **Obinna A. Osuchukwu:** Writing – review & editing, Formal analysis, Writing – review & editing. **Jimoh Abass:** Data curation, Formal analysis. **Christian Ogwuche:** Writing – review & editing, Data curation. **Naresh D. Bansod:** Writing – review & editing, Formal analysis. **Michael I. Ubgaja:** Writing – review & editing, Formal analysis. **Ibrahim Umar:** Writing – review & editing, Formal analysis. **Bello Abdulkareem:** Investigation. **Rabiu K. Olawale:** Writing – review & editing. **Luqman K. Abidoye:** Writing – review & editing.

## Declaration of competing interest

The authors declare that they have no known competing financial interests or personal relationships that could have appeared to influence the work reported in this paper.

## Abbreviations

LDPE: Low-Density Polyethylene
HCl: Hydrochloric acid
XRD: X-ray Diffraction
HV: Vickers Pyramid Number
NaOH: Sodium Hydroxide
wt%: Weight Percentage
M: Molar concentration
pH: potential of Hydrogen
kV: Kilo-voltage
mA: milliampere
SEM: Scanning Electron Microscopy
ICCD: International Centre for Diffraction Data
K_lc_: Fracture toughness
Eqs: Equation
GPa: Giga Pascal
MPa: Mega Pascal
CoF: Coefficient of Friction
